# Surgical correction of septal deviation after Le Fort I osteotomy

**DOI:** 10.1186/s40902-016-0067-z

**Published:** 2016-05-04

**Authors:** Young-Min Shin, Sung-Tak Lee, Tae-Geon Kwon

**Affiliations:** 1 Department of Dentistry and Oral Surgery, Dong-San Medical Center, Keimyung University, 194, Dong-San Dong, Jung Gu, Taegu City, 700-712 South Korea; 2 Center for Orthognathic surgery, Department of Oral and Maxillofacial Surgery, School of Dentistry, Kyungpook National University, Samduck 2 Ga, Jung Gu, Daegu, 700-421 South Korea

**Keywords:** Nasal septum, Le Fort I, Osteotomy

## Abstract

**Background:**

The Le Fort I osteotomy is one of the most widely used and useful procedure to correct the dentofacial deformities of the midface. The changes of the maxilla position affect to overlying soft tissue including the nasal structure. Postoperative nasal septum deviation is a rare and unpredicted outcome after the surgery. There are only a few reports reporting the management of this complication.

**Case Presentation:**

In our department, three cases of the postoperative nasal septum deviation after the Le Fort I osteotomy had been experienced. Via limited intraoral circumvestibular incision, anterior maxilla, the nasal floor, and the anterior aspect of the septum were exposed. The cartilaginous part of the nasal septum was resected and repositioned to the midline and the anterior nasal spine was recontoured. Alar cinch suture performed again to prevent the sides of nostrils from flaring outwards. After the procedure, nasal septum deviation was corrected and the esthetic outcomes were favorable.

**Conclusion:**

Careful extubation, intraoperative management of nasal septum, and meticulous examination of pre-existing nasal septum deviation is important to avoid postoperative nasal septum deviation. If it existed after the maxillary osteotomy, septum repositioning technique of the current report can successfully correct the postoperative septal deviation.

## Background

The Le Fort I osteotomy is the most popular and useful method to correct the dentofacial deformities of the midface. However, the soft tissue changes may have existed after the maxillary osteotomy: the changes of width of the alar base, upturning of the nasal tip, upper lip thinning, downturning of the commissures, and opening of the nasolabial angle [1, 2]. The changes of the maxillary position also can influence the overlying soft tissue including the nasal structure [3]. Surgeons must recognize the potential changes that can occur to the nasal structure and sometimes may plan additional procedures to correct the changes in nasal structure. Especially, in cases with maxillary impaction osteotomy, positional changes of the maxilla can influence the septum and can change the perinasal architecture. Therefore, nasal septum reduction should be accompanied to prevent postsurgical bucking or deviation of the septum [2]. It had been reported that nasal septum deviation is a rare anatomical complication but one of the obvious complications because it also can cause breathing problems and snoring [4].

In this case report, we report our experience of management of septum deviation after maxillary superior impaction, advancement, or canting correction and reviewed the possible causes and treatment of postoperative septal deviation.

## Case presentation

### Case 1

**Fig. 1:**
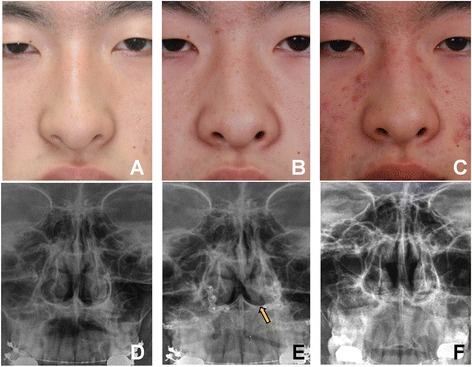
A Facial photographs and cephalo PA radiograph of the patient (case 1) before Le Fort I osteotomy (**a**, **d**), immediately after the maxillary osteotomy that showed the deviation of the septum to the left side (**b**, **e**). The *arrow* indicates the caudal part of the septal cartilage (**e**). After the surgical correction of nasal septum deviation (**c**, **f**)

An 18-year-old man presented with vertical excess and mandibular prognathism without maxillary asymmetry (Fig. 1a, d). The patient underwent a maxillary posterior impaction (5 mm, at maxillary first molar) and advancement at A point (5 mm) and bilateral sagittal split ramus osteotomy (BSSRO) setback (9 mm on the right side, 10 mm on the left). Alar base cinch suture had been performed. At 2 months after the operation, postoperative swelling subsided completely. The patient complained asymmetry and distortion of the nasal septum and nasal bridge (Fig. 1b). Radiographic findings revealed that the nasal septum deviated to the left side compared to the anterior nasal spine (ANS) (Fig. 1e). The patient wanted to have septal correction at the time of plate removal under the general anesthesia. At 1 year after the initial surgery, plate removal and septal correction had been performed under orotracheal intubation. After the procedure, nasal septum deviation was corrected and the patient was satisfied with the outcome of the septal correction (Fig. 1c, f).

**Fig. 2:**
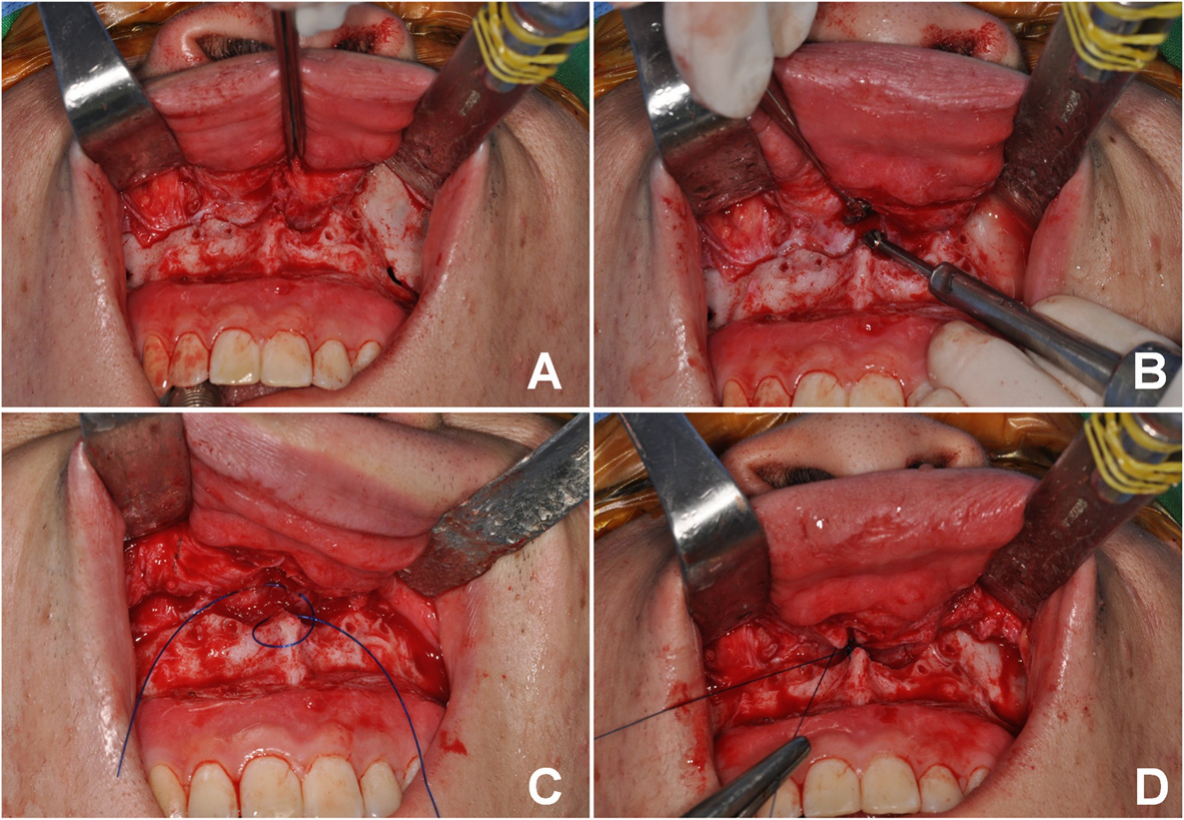
Surgical technique for correction of septal deviation after LeFort I osteotomy. **a** Anterior maxilla, the nasal floor, and the anterior aspect of the septum were visualized. **b** Anterior nasal spine (ANS) was recontoured. **c** Figure-8 style suture to secure the position of the septal cartilage to the ANS. **d** Alar base cinch suture to minimize alar base widening

The surgical technique is demonstrated in Fig. 2. A limited intraoral circumvestibular incision was made to dissect the anterior maxilla, the nasal floor, and the anterior aspect of the septum. The deviated quadrangular cartilage was visualized (Fig. 2a). The ANS was vertically and horizontally recontoured by round bur (Fig. 2b). The nasal septum was resected and repositioned to the midline correctly. A small drill hole was made in ANS and the cartilageonous nasal septum was sutured and tied to the drill hole in ANS by using the figure-8 suture with 3-0 prolene (Fig. 2c). Additionally, alar base cinch suture was made to bring back the surrounding muscle together and prevent the nostril from flaring after the surgery. The method was similar to the previous report by Shojii [5]. The fibroareolar tissue and perinasal muscle were sutured just beneath the skin and alar cartilage. Bilateral tissues were repositioned medially with a large, curved needle (2-0 prolene) (Fig. 2d).

### Case 2

**Fig. 3:**
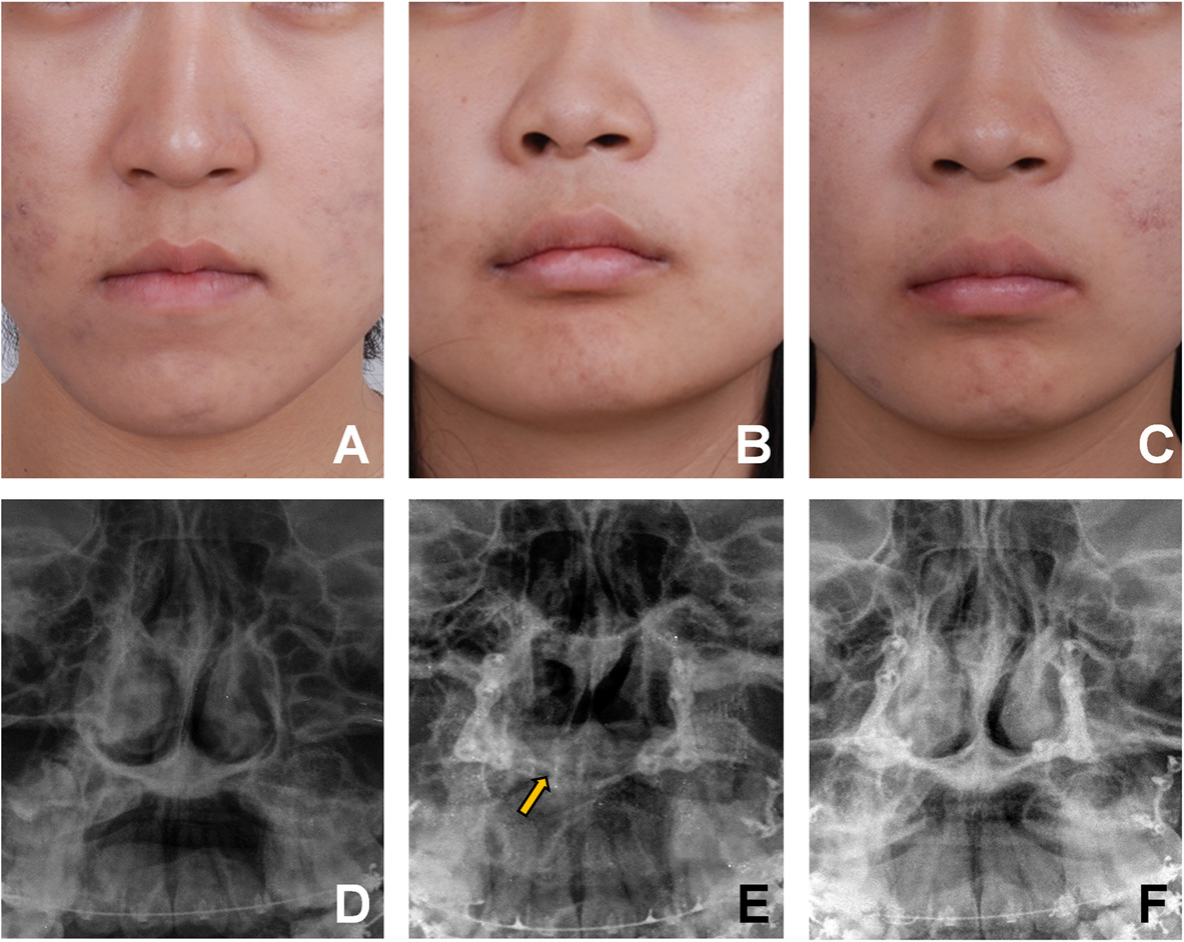
Presurgical condition of case 2 patient before Le Fort I osteotomy (**a**, **d**), immediate after the Le Fort I osteotomy (**b**). Deviated nasal septum (*arrow*) was visible in radiographic examination (**e**). After correction of nasal septum deviation (**c**, **f**). The patient showed corrected septal deviation after the treatment

A 19-year-old woman presented with mandibular prognathism, anterior open-bite, maxillary canting with mandibular deviation to the left side (Fig. 3a, d). The patient had the pre-existing septal deviation before surgery. The maxilla was rotated clockwise with canting correction (1 mm anterior downward; posterior impaction 3 mm on the right side, 1 mm on the left side), BSSRO (right 3 mm, left 11 mm setback), and advancement genioplasty. At 2 weeks after the operation, significant asymmetry and distortion of the base of the nose and nasal tip were recognized (Fig. 3b, e). Nasal septum and dorsum was deviated to the right side. The correction of the septum deviation was performed under local anesthesia. Surgical procedure was similar to previous cases. Deviated nasal bridge and septal bucking disappeared after the secondary surgery (Fig. 3c, f).

### Case 3

**Fig. 4:**
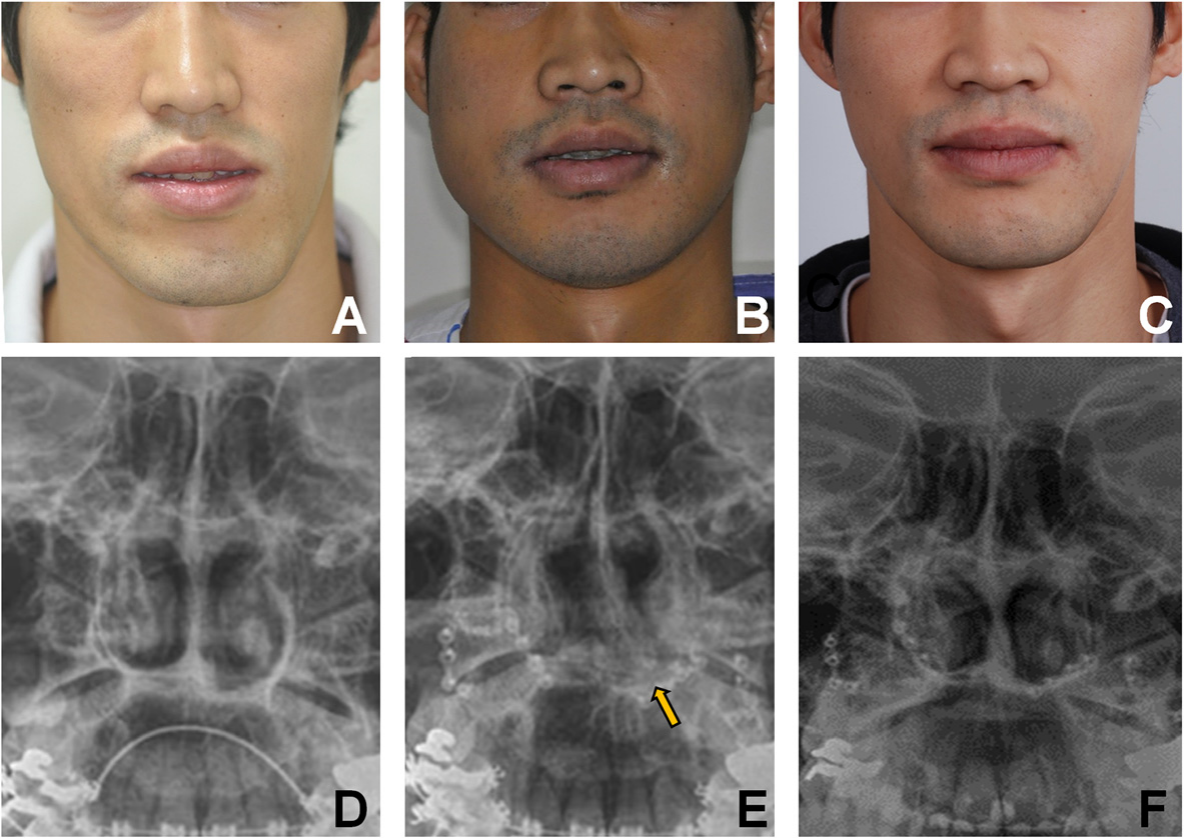
Clinical and radiographic findings of the case 3 patient before Le Fort I osteotomy (**a**, **d**), immediate after Le Fort I osteotomy (**b**, **e**), The *arrow* indicates the caudal part of the septal cartilage (**e**). Sepal deviation was successfully resolved after the corrective surgery (**c**, **f**)

A 26-year-old man presented with mandibular prognathism and mandibular deviation to the left side (Fig. 4a, d). The patient underwent a Le Fort I (bilateral 5 mm posterior impaction, anterior movement of the ANS point to 5 mm) and BSSRO (right 8.5 mm and left 0 mm setback). The patient underwent alar base cinch suture during the surgery. At 5 days after the surgery, postoperative swelling subsided and significant asymmetry and distortion of the nasal base and tip were recognized. Nasal septum deviation was confirmed by clinical and radiographic examination. The nasal septum deviated to the left side (Fig. 4b, e). The deviated septum caused bucking of the septum on the left nasal cavity. The day we recognized the nasal septum deviation, the immediate correction was performed under local anesthesia. The surgical technique was similar to patient #1. After the procedure, nasal septum deviation was corrected and recovery was favorable (Fig. 4c, f).

### Discussion

The Le Fort I osteotomy has become one of versatile procedures in orthognathic surgery during the last decades, mostly making a satisfactory result. However, the Le Fort I osteotomy is still a complex surgery because the maxilla can move in any plane and vector which causes the skeletal changes that affect the position of various anatomical landmarks [6]. The possibility of the alterations in nasal morphology after the Le Fort I osteotomy is well documented in various publications [4, 7]. Based on the consecutive experience of the 1000 cases of Le Fort I osteotomies, Kremer et al. reported that deviation of the nasal septum was observed in 1.6 % patients and were noticed in a few days after surgery when the swelling subsides [4]. In the previous report, postoperative septal deviation after the maxillary surgery may result in breathing problems and snoring. However, our patients did not experience such symptoms. Nasal septum deviation is not the most common reason for persistent postoperative nasal obstruction but can be a secondary cause of postoperative persistent nasal obstruction with inadequate recontouring of the nasal floor, the anterior nasal spine, and the pyriform rims.

The chief complaints of our patients were the asymmetry of nose and crooked shape of nose. Postoperative septal deviation after Le Fort I surgery would be attributed to the following causes. At first, if the septum was not resected similarly with a degree of impaction, it can cause a buckled, deviated septum by increased maxillary bony support if the surgeon needs to resect the inferior aspect of the septum or superior aspect of ANS when finishing maxillary superior impaction. However, reduction or recontouring of ANS is discouraged in patients with poor nasal tip projections or maxillary setback procedures [7]. Especially in oriental patients, most of the patients have weak nasal projection and it is not indicated to perform overzealous reduction of ANS. When the maxillary advancement is indicated, protruding ANS also can cause the bucking of the nasal cartilage. Second, the dissected periosteum and muscle adjacent to the nose were not replaced adequately. If these muscles were not brought back together in conjunction with the intraoral mucosal closure, the sides of the nostrils will flare outwards. The third reason is altered position or shape of the anterior nasal spine and postsurgical edema. However, there is not only intraoperative cause but also perioperative cause, it is usually due to the lack of clear vision of surgical field and restricted range of movement of the oro-cranial base during a Le Fort I osteotomy because of the nasotracheal intubation. This intubation method makes it difficult to estimate the amount of reduction in nasal septum and causes the nasal septum deviation [8, 9]. If the nasal septum is properly trimmed at operation, the deviation of the septum after the nasotracheal tube removal should be uncommon. Another possible reason for septum deviation is dislocation of the quadrangular cartilage by a incomplete deflated cuff during extubation. Manual examination of the nose after extubation is important but often neglected. Visual inspection of the nose is important after repositioning of the maxilla before and after extubation to confirm the intact position of septum. If displacement of septum is detected after extubation and is generally due to nasotracheal tube pressure, then limited manual manipulation in the recovery room would be available [10].

**Fig. 5:**
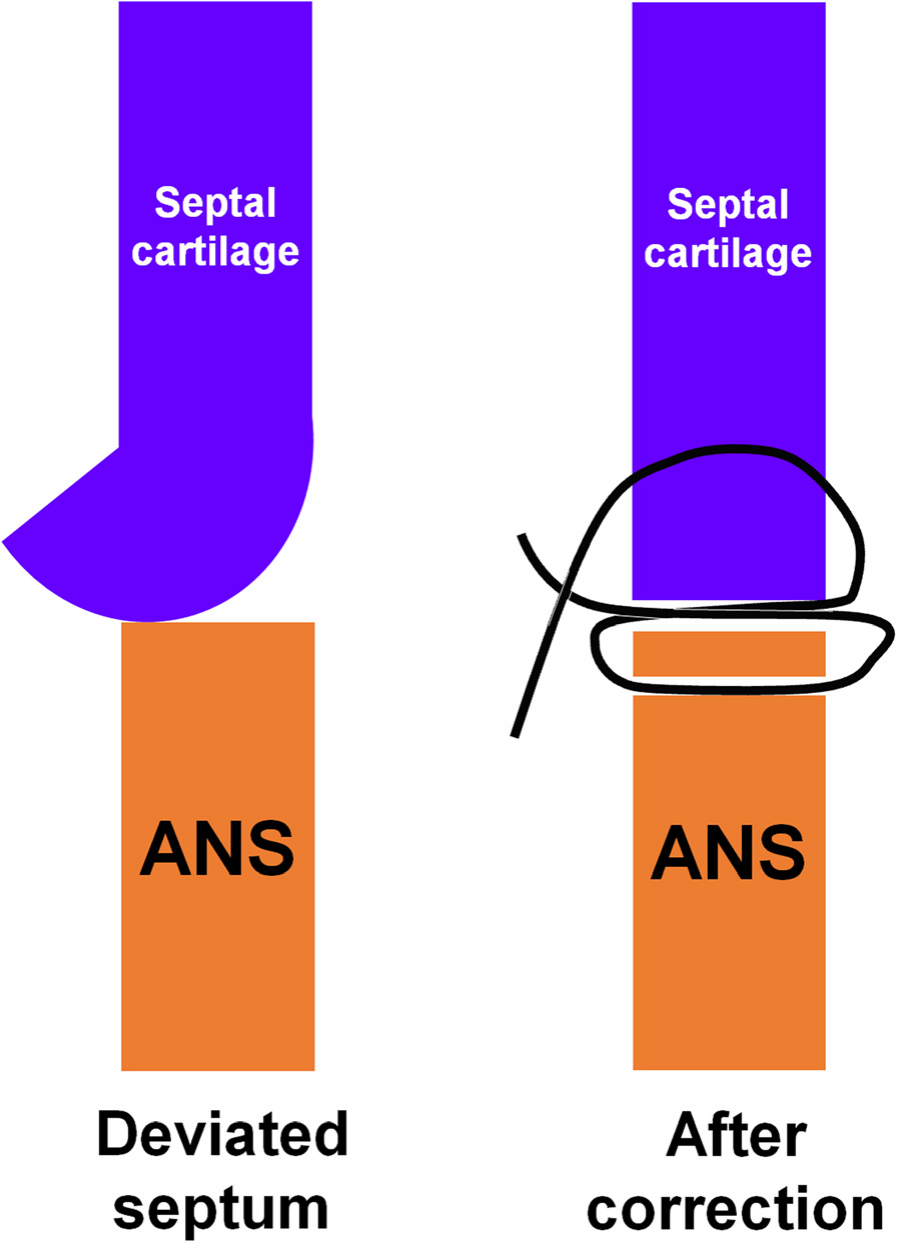
Schematic drawing of the septal cartilage to ANS suture by using the “figure-of-8” suture to stabilize the caudal part of the septal cartilage on the ANS

There is no standard of care for the nasal septum deviation after the surgery. As far as we know, there are only few literatures mentioning the specific surgical methods for the septal deviation. According to Van Sickels [7], there would be three choices for the secondary septal deviation after surgery; immediate manipulation (blind method like nasal bone reduction), immediate re-operation or septoplasty at a later stage when the patients do not have an airway problem. In our experience, immediate postsurgical manipulation would not be successful especially when the patients have the postoperative swelling. Furthermore, inadequate repositioning can result in more serious complaints from the patients. Therefore, we went back to the basic, which is a similar approach for the maxillary impaction Le Fort I osteotomy; additional reduction of septum, alar cinch suture, and reduction of the anterior nasal spine. Especially, the caudal part of the septal cartilage was tied with figure-8 suture technique and secured in position above the ANS by using a small hole (Fig. 5). This technique can have advantages: (1) corrected position of the septum can be tightly secured in position even after the extubation procedure, and (2) anatomical repositioning can minimize the shape changes in columella. To prevent septum deviation, same or more than 3 mm septum reduction as a degree of maxillary impaction was recommended [2].

The resection of the deviated portions of the septum at the time of Le Fort I osteotomy would be also beneficial for breathing and sinus drainage. The recontouring of the anterior nasal spine is indicated when the patients have preoperative good nasal tip projection and need to perform large advancement or impaction of the maxilla. Patients also have constricted and asymmetric pyriform rims and nasal floor and deviated anterior nasal spine; thus, after Le Fort I osteotomy, the recontouring of these areas are necessary to open the airway and improve nasal esthetics. Alar cinch suture is often suggested to control the nasal soft-tissue envelope and indicated when reduction of the anterior extent of the piriform rim, the anterior nasal spine, and trimming of the height of anterior nasal floor. The alar cinch suture is utilized to prevent nasal base widening after undergoing advancement and/or superior impaction of the maxilla. The alar base width is measured with calipers and the measurement recorded before the beginning of surgery. The alar base width is again measured postoperatively and compared with the preoperative value to ensure that proper surgical control of the alar base width has been maintained [11].

## Conclusions

Postoperative nasal septum deviation can be caused by insufficient reduction of the septum when performing maxillary impaction, incorrect bringing back of muscle adjacent to the nose, altered position and/or shape of the anterior nasal spine, postoperative swelling, and nasotracheal intubation. Precise surgical skill, careful extubaction, and meticulous examination of the nose are very important to avoid nasal septum deviation. If it exists after the surgery, septal cartilage reduction/repositioning, ANS recontouring, and alar base cinch suture can be used to solve these problems. The surgical method that is used in our case series can be a suitable method for secondary correction of the septal deviation. This surgical method also can be used intraoperatively and can minimize the potential deviation of the septal cartilage after maxillary surgery.

## Consent

Written informed consent was obtained from the patient for the publication of this report and any accompanying images.
